# Optimal Duration of Follow-up for Assessing Antimalarial Efficacy in Pregnancy: A Retrospective Analysis of a Cohort Followed Up Until Delivery on the Thailand–Myanmar Border

**DOI:** 10.1093/ofid/ofz264

**Published:** 2019-05-31

**Authors:** Makoto Saito, Rashid Mansoor, Jacher Wiladphaingern, Moo Kho Paw, Mupawjay Pimanpanarak, Stephane Proux, Philippe J Guérin, Nicholas J White, François Nosten, Rose McGready

**Affiliations:** 1 Shoklo Malaria Research Unit (SMRU), Mahidol-Oxford Tropical Medicine Research Unit, Faculty of Tropical Medicine, Mahidol University, Mae Sot, Tak, Thailand; 2 Centre for Tropical Medicine and Global Health, Nuffield Department of Medicine, University of Oxford, Oxford, UK; 3 WorldWide Antimalarial Resistance Network (WWARN); 4 Mahidol–Oxford Tropical Medicine Research Unit (MORU), Faculty of Tropical Medicine, Mahidol University, Bangkok, Thailand

**Keywords:** duration of follow-up, efficacy, malaria, *Plasmodium falciparum*, pregnancy

## Abstract

**Background:**

Follow-up for 28–42 days is recommended by the World Health Organization to assess antimalarial drug efficacy for nonpregnant populations. This study aimed to determine the optimal duration for pregnant women, as no specific guidance currently exists.

**Methods:**

The distributions of time to recrudescence (treatment failure), confirmed by polymerase chain reaction genotyping for different antimalarial drugs in pregnancy, were analyzed by accelerated failure time models using secondary data on microscopically confirmed recurrent falciparum malaria collected in prospective studies on the Thailand–Myanmar border between 1994 and 2010.

**Results:**

Of 946 paired isolates from 703 women, the median duration of follow-up for each genotyped recurrence (interquartile range) was 129 (83–174) days, with 429 polymerase chain reaction–confirmed recrudescent. Five different treatments were evaluated, and 382 *Plasmodium falciparum* recrudescences were identified as eligible. With log-logistic models adjusted for baseline parasitemia, the predicted cumulative proportions of all the recrudescences that were detected by 28 days were 70% (95% confidence interval [CI], 65%–74%) for quinine monotherapy (n = 295), 66% (95% CI, 53%–76%) for artesunate monotherapy (n = 43), 62% (95% CI, 42%–79%) for artemether–lumefantrine (AL; n = 19), 46% (95% CI, 26%–67%) for artesunate with clindamycin (n = 19), and 34% (95% CI, 11%–67%) for dihydroartemisinin–piperaquine (DP; n = 6). Corresponding figures by day 42 were 89% (95% CI, 77%–95%) for AL and 71% (95% CI, 38%–91%) for DP. Follow-up for 63 days was predicted to detect ≥95% of all recrudescence, except for DP.

**Conclusions:**

In low-transmission settings, antimalarial drug efficacy assessments in pregnancy require longer follow-up than for nonpregnant populations.

Effective antimalarial medicines are required to limit the adverse effects of malaria in pregnancy, which are deleterious to both the mother and fetus. Global implementation of highly efficacious artemisinin-based combination therapies (ACT) has changed the treatment of *Plasmodium falciparum* malaria dramatically [1]. The efficacy of antimalarial drugs needs to be monitored because of the continued emergence and/or spread of resistance. The World Health Organization (WHO) recommends that antimalarial policy be changed if the efficacy of treatments is ≤90% [2]. A declining trend in efficacy over time therefore requires closer monitoring, particularly in vulnerable populations such as pregnant women.

According to the current WHO guidelines, in evaluating the efficacy of antimalarial treatment, patients should be followed up actively with clinical and parasitological assessments by microscopy to detect recurrence of *P. falciparum* malaria [3]. Recurrence of parasitemia can be either recrudescence of the same parasite “strain” that caused the initial infection (ie, treatment failure) or reinfection with a new strain. Provided there is sufficient genetic diversity in the prevailing parasite populations, this is distinguished by polymerase chain reaction (PCR) genotyping of polymorphic loci [4]. The currently recommended duration of follow-up for nonpregnant populations is a minimum of 28 days (for regimens containing amodiaquine, artemisinin derivatives, chloroquine, lumefantrine, quinine, or sulfadoxine–pyrimethamine) to 42 days (for piperaquine and mefloquine) [3].

Currently, there is no standardized guideline for assessing antimalarial efficacy in pregnancy, and the guideline for nonpregnant patients is frequently adopted [5, 6]. Both the efficacy and the optimal duration of follow-up could be different in pregnant women because of altered immunity, physiological changes affecting antimalarial pharmacokinetics, and a unique parasite haven in the placenta [5, 7–10]. It seems that pregnant women can harbor blood stage infections for protracted periods; previous studies have reported recrudescences >100 days after treatment in pregnant women both in high-transmission areas in sub-Saharan Africa [11–13] and in lower-transmission areas in South East Asia [8, 14].

The objective of this study was to characterize the factors that affect the time to recrudescence of *P. falciparum* infections in pregnancy in a cohort followed until delivery and to predict what proportion of recrudescent infections would be detected by days 28, 42, and 63 for each treatment, after adjusting for other risk factors.

## METHODS

### Study Settings

This study included secondary data collected prospectively in treatment efficacy studies on uncomplicated falciparum malaria in pregnancy [7, 8, 14–21]. The studies included were all conducted on the Thailand–Myanmar border, where the transmission of falciparum malaria is low and unstable. In this setting, pregnant women presenting to antenatal clinics were screened for malaria by checking peripheral blood smear by microscopy every 1–2 weeks regardless of their symptoms. Sulfadoxine–pyrimethamine chemoprevention was not adopted in this area due to the very high resistance reported 4 decades ago [22], and intermittent preventive treatment in pregnancy is not recommended in low-transmission settings. The pregnant women actively detected in this way were enrolled into prospective treatment studies, provided they gave fully informed consent, and were then followed up with malaria screening by microscopy until delivery (or 42–63 days, whichever came later). Treatment was given under direct observation, and the doses used in these studies were within the WHO-recommended ranges [2]. PCR genotyping (based on polymorphic segments of *msp1*, *msp2*, and *glurp*) was done for *P. falciparum* recurrences regardless of the intervening interval or any intercalated *Plasmodium vivax* infections. If all 3-locus genotypes were the same before and after the treatment, the recurrence was considered a recrudescence [4]. The reliability of the use of these 3 molecular markers in this area has been validated previously [23]. This analysis uses data collected between 1994 and 2010, before widespread artemisinin resistance started to be observed in this area [24, 25].

### Inclusion Criteria

Recurrent malaria episodes (defined here as the reappearance of peripheral falciparum malaria parasitemia detected by microscopy regardless of the symptoms) confirmed by PCR genotyping as recrudescence in pregnant women in any trimester who completed a quinine- or artemisinin-based treatment were included in the analysis. Only late treatment failure (ie, recurrence of parasitemia on or after day 7) [3], which requires PCR genotyping, was included. Treatment groups with <5 recrudescent infections were excluded from the analyses.

### Modeling the Distribution of Time to Recrudescence

For each treatment group, baseline characteristics were described as median and range for continuous variables and as proportions for categorical variables.

To model the time to treatment failure, episodes of PCR-confirmed recrudescence were analyzed by accelerated failure time (AFT) models (namely, exponential, Weibull, log-normal, and log-logistic distributions). These were compared without any covariates using the Akaike information criterion (AIC) [26].

The covariates with *P *<.05 by the Wald test in univariable analysis were all included in the multivariable model. Two variables, namely treatment group and baseline asexual parasite densities, were always included in the multivariable model as they were known a priori to affect recrudescence risk. *P. vivax* intercalated infections preceding PCR-confirmed *P. falciparum* recrudescence were included in the analysis but regarded as being censored on the day of *P. vivax* infection. A sensitivity analysis without censoring of *P. vivax* infections was also conducted. Each episode was regarded as unique in the analysis. A complete case analysis was conducted.

As a sensitivity analysis, flexible parametric models with 0–2 knots were constructed [27], but the model without knots was selected as the final model because of the simplicity and interpretability; only the model without knots can be interpreted as an accelerated failure time model. Another sensitivity analysis was conducted including mefloquine monotherapy, which is neither an artemisinin-based nor quinine-based treatment.

Cumulative probability densities with commonly used follow-up durations (ie, days 28, 42, and 63) were predicted using the final model. Probability density curves based on the final model were drawn for each drug to assess the distribution of recrudescence over time. Statistical analyses were done using R (R Foundation for Statistical Computing, Vienna, Austria) and Stata MP 15.1 (StataCorp, College station, TX, US).

### Ethics

Ethics approval for retrospective analysis of anonymized secondary data at SMRU was granted by the Oxford Tropical Research Ethics Committee (OxTREC 28-09) and the Tak Community Advisory Board (TCAB-4/1/2015).

## RESULTS

### Demographic Information

**Figure 1.**
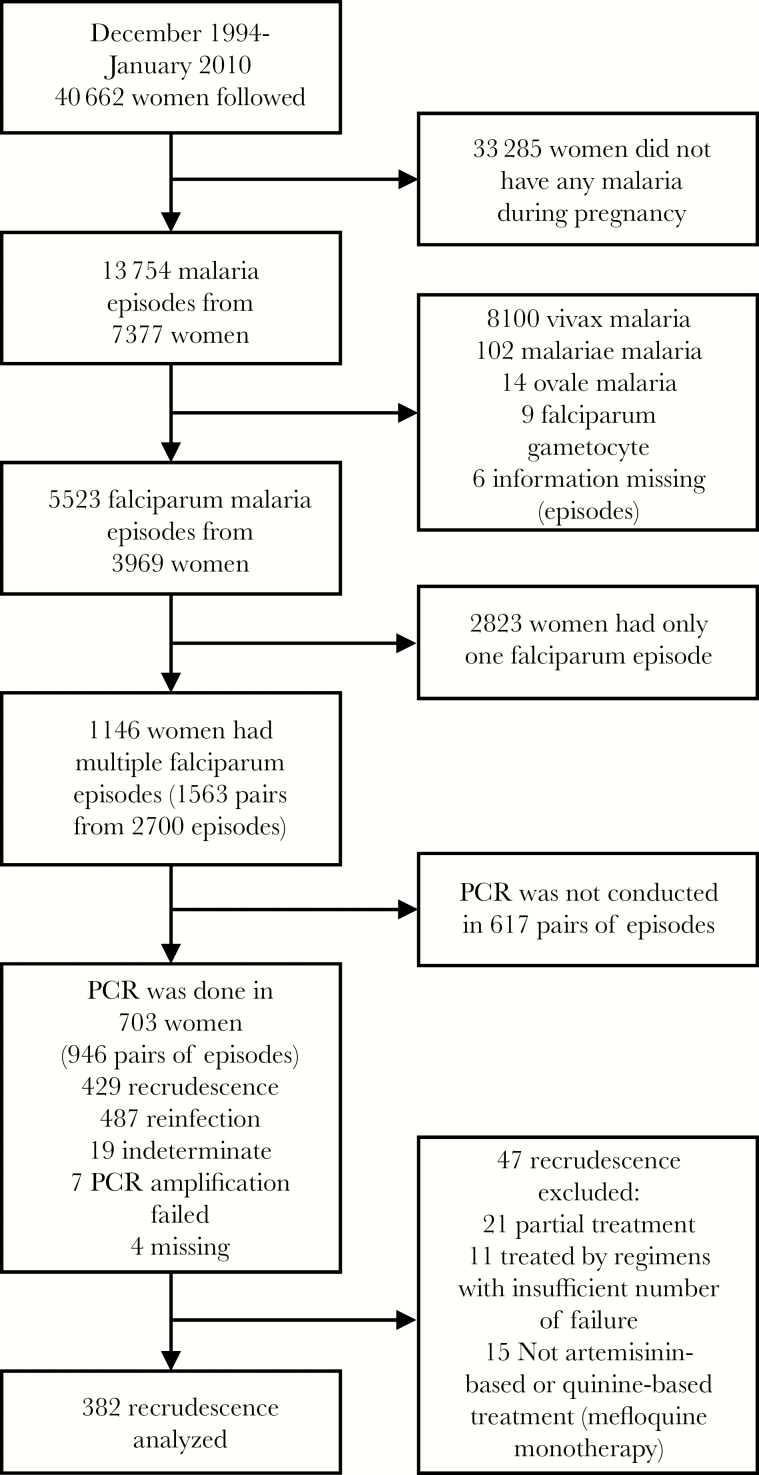
Number of recurrent malaria episodes in pregnant women attending antenatal care on the Thailand–Myanmar border, 1994–2010. Abbreviation: PCR, polymerase chain reaction.

Of 40 662 pregnant women followed in this prospective cohort between December 1994 and January 2010, 3969 (10%) had at least 1 episode of falciparum malaria (Figure 1). During a median (interquartile range [IQR]) of 97 (37–160) days of follow-up, 1146 women had more than 1 episode of falciparum malaria. Among these recurrences, 946 paired isolates from 703 women were PCR genotyped. The median duration of follow-up for each genotyped recurrence was 129 (IQR, 83–174) days. There were 429 PCR-confirmed recrudescent infections, and 382 eligible recrudescent episodes in 341 women were included in the analysis. Five antimalarial treatments were included in this analysis: quinine monotherapy (n = 295), artesunate monotherapy (AS; n = 43), artemether–lumefantrine (AL; n = 19), artesunate with clindamycin (AC; n = 19), and dihydroartemisinin–piperaquine (DP; n = 6).

Of the 382 episodes, 357 (93%) were *P. falciparum* mono infections and the remainder were mixed infections with *P. vivax* (Table 1). The geometric mean parasite density (range) was 4287 (32–422 016) asexual parasites/µL. Because of the active screening, only 31% (105/344) of women were febrile at presentation (>37.5ºC), although 73% (274/373) reported a history of fever in the last 48 hours before admission.

**Table 1.** Baseline Characteristics of Pregnant Women With Uncomplicated Falciparum Malaria

	Quinine (n = 295)		AS (n = 43)		AL (n = 19)		AC (n = 19)		DP (n = 6)	
Baseline Characteristic	No. (%)	Median (Range)	No. (%)	Median (Range)	No. (%)	Median (Range)	No. (%)	Median (Range)	No. (%)	Median (Range)
Age, y										
<20	65 (22)		9 (21)		2 (11)		7 (37)		1 (17)	
20–24	75 (25)		8 (19)		4 (21)		7 (37)		0	
25–29	87 (29)		11 (26)		5 (26)		1 (5)		1 (17)	
≥30	68 (23)		15 (35)		8 (42)		4 (21)		4 (67)	
Gravidity										
1	85 (29)		9 (21)		3 (16)		9 (47)		1 (17)	
2	67 (23)		8 (19)		5 (26)		1 (5)		0	
≥3	143 (48)		26 (60)		11 (58)		9 (47)		5 (83)	
Trimester										
1st trimester	99 (34)		1 (2)		0		2 (11)		0	
2nd trimester	157 (53)		28 (65)		10 (53)		10 (53)		5 (83)	
3rd trimester	39 (13)		14 (33)		9 (47)		7 (37)		1 (17)	
Weight, kg	295	46 (32–77)	43	46 (36–62)	19	50 (40–65)	19	49 (31–58)	6	52.5 (47–57)
Fever (>37.5°C)										
Yes	81 (27)		13 (30)		6 (32)		4 (21)		1 (17)	
No	179 (61)		27 (63)		13 (68)		15 (79)		5 (83)	
Missing	35 (12)		3 (7)		0		0		0	
Parasitemia, log_10_/µL	295	3.6 (1.5–5.3)	43	4.0 (1.5–5.6)	19	4.2 (2.1–5.2)	19	3.1 (1.5–5.3)	6	4.2 (3.3–5.1)
Species										
Falciparum only	276 (94)		38 (88)		19 (100)		18 (95)		6 (100)	
Mixed infection	19 (6)		5 (12)		0		1 (5)		0	
Previous antimalarial treatment in 28 d										
Yes	45 (15)		19 (44)		11 (58)		7 (37)		4 (67)	
No	250 (85)		24 (56)		8 (42)		12 (63)		2 (33)	

Abbreviations: AC, artesunate with clindamycin; AL, artemether–lumefantrine; AS, artesunate monotherapy; CI, confidence interval; DP, dihydroartemisinin–piperaquine.

There was no apparent difference in baseline characteristics among the treatment groups except for estimated gestational age and history of antimalarial use (Table 1). Despite its poor efficacy and tolerability profile, quinine is still the drug of choice recommended by the WHO in the first trimester of pregnancy, so the estimated gestational age was lower, and fewer patients had antimalarial treatment in the previous 28 days in the quinine group. Artemisinin-based treatments (AC, AL, AS, and DP) were used as either the firstline (in the second or third trimester) or rescue treatment for quinine failures.

### Factors Affecting the Time to Recrudescence

**Figure 2.**
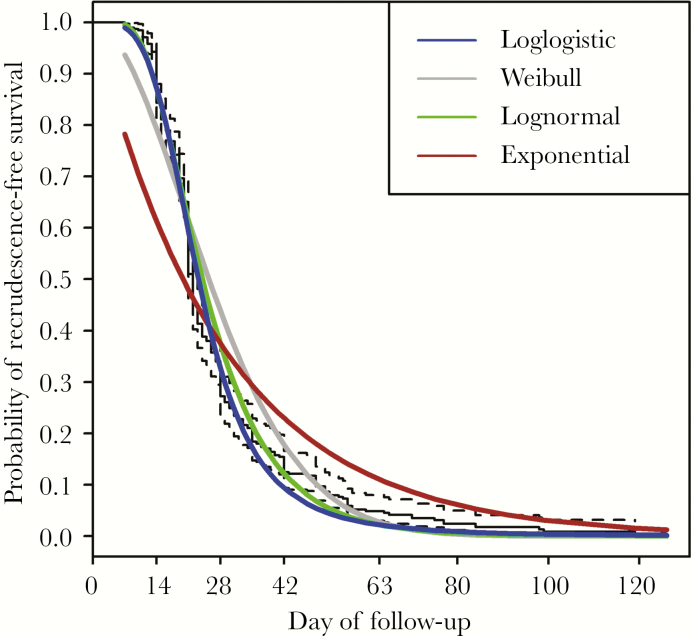
Recrudescence-free survival curves in pregnant women treated for uncomplicated falciparum malaria on the Thailand–Myanmar border, 1994–2010. Ninety-five percent confidence intervals using Kaplan-Meier estimates are shown with dotted lines.

Among the different AFT models, a log-logistic model was chosen for the final analysis (Figure 2). In the univariable analysis, treatment was associated with time to recrudescence (Table 2). With quinine as the reference, the interval to recrudescence was 1.04 (95% confidence interval [CI], 0.90–1.20) times longer after AS, 1.08 (95% CI, 0.87–1.35) times after AL, 1.30 (95% CI, 1.03–1.63) times after AC, and 1.46 (95% CI, 1.02–2.08) times after DP. Although the presence of fever and *P. vivax* co-infection showed shorter times to recrudescence, neither fulfilled the inclusion criteria for multivariable models (*P *< .05).

**Table 2.** Univariable and Multivariable Analyses for the Time to Recrudescence in Pregnant Women by Log-Logistic Accelerated Failure Time Models

	Univariable Analysis		Multivariable Analysis	
Baseline Characteristic	Acceleration Factor (95% CI)	*P* Value	Acceleration Factor (95% CI)	*P* Value
Age				
<20 y	Reference			
20–24 y	0.90 (0.78–1.03)	.12		
25–29 y	0.92 (0.80–1.05)	.20		
≥30 y	0.96 (0.84–1.10)	.56		
Gravidity				
1	1.05 (0.94–1.17)	.38		
2	0.99 (0.88–1.10)	.80		
≥3	Reference			
Estimated gestational wk	1.00 (0.99–1.01)	.98		
Weight, kg	1.00 (1.00–1.01)	.28		
Parasitemia, log_10_/µL	0.98 (0.93–1.03)	.35	0.97 (0.93–1.02)	.29
Fever (>37.5°C)				
Yes	0.96 (0.86–1.06)	.40		
No	Reference			
Mixed infection				
Yes	0.87 (0.73–1.04)	.12		
No	Reference			
Previous antimalarial treatment in 28 d				
Yes	1.00 (0.90–1.12)	.96		
No	Reference			
Treatment				
Quinine	Reference		Reference	
AS	1.04 (0.90–1.20)	.61	1.05 (0.91–1.21)	.54
AL	1.08 (0.87–1.35)	.50	1.09 (0.87–1.36)	.45
AC	1.30 (1.03–1.63)	.03	1.29 (1.03–1.62)	.03
DP	1.46 (1.02–2.08)	.04	1.48 (1.03–2.11)	.03

Abbreviations: AC, artesunate with clindamycin; AL, artemether–lumefantrine; AS, artesunate monotherapy; CI, confidence interval; DP, dihydroartemisinin–piperaquine.

In the multivariable analysis, the type of treatment was associated with time to recrudescence adjusted by baseline parasitemia. The adjusted interval to recrudescence compared with that after quinine was 1.05 (95% CI, 0.91–1.21) times longer after AS, 1.09 (95% CI, 0.87–1.36) times after AL, 1.29 (95% CI, 1.03–1.62) times after AC, and 1.48 (95% CI, 1.03–2.11) times after DP.

### Distribution of Intervals to Recrudescence

**Figure 3.**
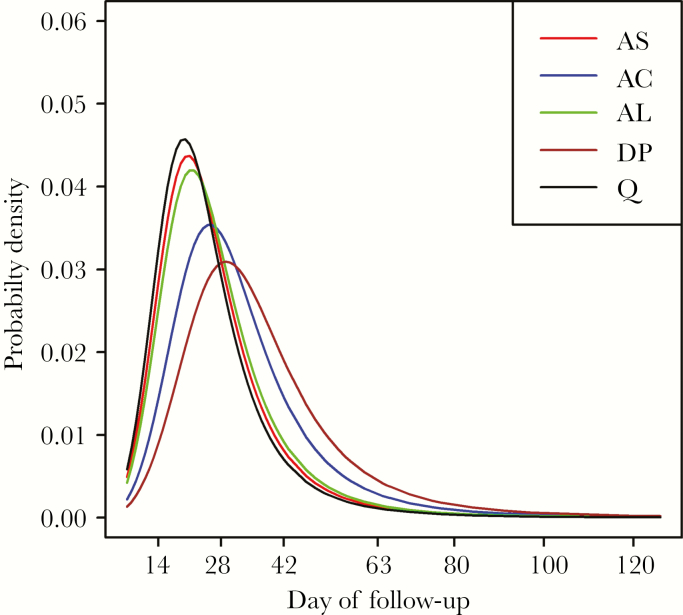
Adjusted probability of recrudescence over time by treatment in pregnant women on the Thailand–Myanmar border, 1994–2010. The mean value in log_10_ scale (4186/µL) is used for baseline parasitemia. Abbreviations: AC, artesunate with clindamycin; AL, artemether–lumefantrine; AS, artesunate monotherapy; DP, dihydroartemisinin–piperaquine; Q, quinine monotherapy.

Based on the final multivariable model, the predicted probability density functions (Figure 3) and the predicted cumulative probabilities of recrudescence (Supplementary Figure 1) were calculated assuming the mean value of the baseline parasitemia on the log_10_ scale. The modal interval to recrudescence was 20 days for quinine, 21 for AS and AL, 25 for AC, and 29 days for DP.

The proportion (cumulative probability) of total treatment failures detected by the end of currently recommended follow-up durations was derived from the final model (Table 3). The predicted cumulative proportion of treatment failures detected was low if the follow-up was only for 28 days: 70% (95% CI, 65%–74%) for quinine, 66% (95% CI, 53%–76%) for AS, 62% (95% CI, 42%–79%) for AL, 46% (95% CI, 26%–67%) for AC, and 34% (95% CI, 11%–67%) for DP. Similarly, ≥10% treatment failures would not be detected if the follow-up duration was 42 days. Follow-up for 63 days was predicted to detect ≥95% of the treatment failures except for DP, for which it was predicted that 92% (95% CI, 74%–98%) of treatment failures would be detected. To detect 95% of all treatment failures in pregnancy, it was estimated that 53 (95% CI, 41–65) days of follow-up were required for AL and 72 (95% CI, 46–97) days for DP.

**Table 3.** Predicted Cumulative Proportions of Treatment Failure Detected by Days 28, 42, and 63 After Antimalarial Treatment in Pregnant Women

	Predicted Cumulative Proportion of Treatment Failure Detected by Different Times (95% CI)		
Treatment	Day 28	Day 42	Day 63
Quinine	0.70 (0.65–0.74)	0.92 (0.89–0.94)	0.98 (0.97–0.99)
AS	0.66 (0.53–0.76)	0.90 (0.84–0.94)	0.98 (0.96–0.99)
AL	0.62 (0.42–0.79)	0.89 (0.77–0.95)	0.97 (0.94–0.99)
AC	0.46 (0.26–0.67)	0.80 (0.63–0.91)	0.95 (0.89–0.98)
DP	0.34 (0.11–0.67)	0.71 (0.38–0.91)	0.92 (0.74–0.98)

Abbreviations: AC, artesunate with clindamycin; AL, artemether–lumefantrine; AS, artesunate monotherapy; CI, confidence interval; DP, dihydroartemisinin–piperaquine.

The predicted proportions of *P. falciparum* recrudescences detected by a model without *P. vivax* infection censoring and by a flexible parametric model were similar to the predictions of the final AFT model (Supplementary Data).

Another sensitivity analysis was conducted including mefloquine monotherapy. The adjusted interval to recrudescence was longer by 1.08 (95% CI, 0.86–1.37) times after mefloquine than after quinine. The predictions for other treatments were similar to those by the final AFT model (Supplementary Data).

## DISCUSSION

Highly efficacious treatment can limit the cumulative deleterious impact of recurrent malaria during pregnancy on the mother and fetus. This demands correct assessment of treatment efficacy, which in turn requires an adequate length of follow-up. This large study from an area of low and seasonal transmission suggests that recommended durations of follow-up to assess antimalarial drug efficacy for nonpregnant populations (ie, 28–42 days) are too short for pregnant populations.

The duration of follow-up in a survival analysis needs to be long enough to capture the majority of events (ie, recrudescence in antimalarial efficacy studies) so that appropriate statistical analysis can be performed [26, 28]. This is particularly important when the events are rare, as is the case for ACTs (where treatment failure should be less than 5%), and the study size is small.

Drug elimination rate (assessed conventionally by terminal half-life) is the main factor determining the interval to recrudescence (ie, as it reflects the time during which a drug suppresses parasite growth). Immunity against malaria affects the survival and growth of parasites independently of the antimalarial drug [29]. The pregnant status can affect both drug elimination and immunity [30, 31], presumably enhancing the survival and growth of parasites.

Previous studies on nonpregnant populations, which assumed that 63 days of follow-up captured all recrudescences, concluded that patients should be followed up for at least 28 days for antimalarial drugs with a short half-life (eg, quinine, AS, and AC), for 42 days for antimalarial drugs with an intermediate half-life (eg, AL), and for 63 days for antimalarial drugs with a long half-life (eg, DP, mefloquine) [9, 28].

These recommendations appear insufficient for pregnant women, at least in a low-transmission setting. This study shows that in pregnant women, a 28-day follow-up can miss ≥30% of the recrudescences for quinine or AS. For AL, 11% (95% CI, 5%–23%) of recrudescences are predicted to be missed with 42-day follow-up. For DP, around 10% of recrudescences are predicted to occur after 63 days, although in this cohort this prediction is based on very small observations (n = 6). Under the currently recommended dosing regimens, this study suggests longer follow-up periods, such as 53 (95% CI, 41–65) days for AL and 72 (95% CI, 46–97) days for DP, to capture 95% of all recrudescences in pregnancy. In the context of antenatal care, this length of time is shorter than 1 trimester and should not be too burdensome. This study did not extend to the postpartum period, and the rapidity of reversion to the nonpregnant adult therapeutic response is unknown.

One previous study simulated the time to recrudescence based solely on drug half-life [32]. In that study, after ACT with a partner drug with a half-life of ≤1 week (eg, AL), the peak of recrudescence was predicted to be day 28. In this current analysis, the peak of recrudescence was around day 21 after AL (and 29 after DP). This difference can be explained partly by the shorter drug terminal elimination half-lives of lumefantrine and piperaquine in pregnant compared with nonpregnant women [30]. Compared with the simulated distribution curves based on drug half-lives [32], the distribution curves modeled in this study had a slightly longer distal tail. Immunity, placental sequestration, and relatively low baseline parasitemia due to active detection may contribute to this longer “tail” of recrudescences.

The time to recrudescence was longer in AC than expected from the drug half-lives of artesunate (<1 hour) and clindamycin (2–4 hours). However, AC, AS, and quinine were given over 7 days, whereas other treatments (ACTs) were given over 3 days. Another possibility is that the higher efficacy achieved by adding clindamycin reduced the residual parasite burden. Similarly, an additional analysis including mefloquine monotherapy, which was used when high-grade resistance to mefloquine was already prevalent and well described in the nonpregnant population in this area [33], shows that higher-grade drug resistance shortens the time to recrudescence (Supplementary Data), which is consistent with the mathematical modeling described previously [34]. To detect lower-grade resistance, a longer duration of follow-up may be needed.

This is the first study to use AFT models to analyze the time to treatment failure in malaria. There are several reasons why the AFT model is better suited to this purpose than the Cox proportional hazards model. Biologically, the assumption of the AFT models is more plausible; the distribution of time to recrudescence is shorter (or longer) depending on factors such as the half-life of the drug. Clinically, the AFT models analyze the times to events directly (rather than hazard ratios); thus the interpretation of the output is clinically relevant and meaningful [35]. AFT model prediction is also better than that of the Cox model for the “right-hand” or distal tail of the distribution [27], which is the main research interest in this study. The disadvantage of the conventional parametric model is the model fit, but in our data, the model fit was adequate and the predicted captured proportion by a flexible parametric model (with a better fit) was similar to the figures of the final AFT model.

Despite these advantages, there are also limitations. First, the number of observations included in this analysis was small for all drugs except quinine. However, modeling enables prediction of the temporal distributions for drugs with fewer observations based on the distributions for other drugs with more observations but with a proportional change in the time to events, as discussed above. The effects of covariates (eg, gestational age) might be different for each drug, but this was not assessed fully.

Second, this study was based on the data collected on the Thailand–Myanmar border, where falciparum malaria transmission is low and unstable. This limits generalizability. Nonetheless, available evidence from other studies is scarce [6]. There are 3 other published studies that report follow-up of patients beyond the current framework of follow-up (ie, for more than 63 days) with PCR genotyping for recurrences, but 2 of them did not have enough recrudescences (ie, <5) for analysis [12, 13] and the third followed up patients less frequently (ie, monthly or less) [36]. Recently, more studies in pregnancy followed up patients for 63 days, which is reassuring for assessing the efficacy of drugs with a short half-life (such as AL or quinine), as was shown in this study. However, as the prediction model implied, 63 days may not be long enough for ACTs with a longer half-life partner drug, such as DP. In a recent multicenter trial conducted in 4 sub-Saharan African countries, most of the recrudescences after DP were reported to have occurred during 56–63 days [37], implying the possibility of recrudescence after 63 days’ follow-up. A previous systematic review highlighted the necessity of more antimalarial treatment studies in pregnancy with an adequate length of follow-up [6].

These predictions need to be tested in high transmission areas, although this becomes more complex because of a higher risk of misclassification with genotyping and a higher level of immunity against malaria. In high-transmission areas, concomitant infection with multiple discrete genotypes is common [38] and the rates of reinfection are high [32]. Both lead to a higher probability of misclassification of reinfection as recrudescence, particularly in the late follow-up period, during which the post-treatment prophylactic effect has waned and the risk of reinfection substantially outweighs that of recrudescence. In all areas, this type of study is difficult because of the long follow-up and large sample size needed when studying efficacious treatments. This particular cohort is unique, with a consistent method of data collection over many years, including quality control of microscopy diagnosis, and a long follow-up length (median, ≥100 days), ensuring that nearly all recrudescences were captured.

The possibility of predicting the “true” efficacy of antimalarial treatments by extrapolating the observed efficacy with a shorter follow-up needs cautious consideration. As was implied in this analysis, the shorter the duration of follow-up, the more uncertain the prediction of “true” efficacy becomes. This uncertainty becomes greater if the number of recrudescences is small. Efforts should be made to capture most of the recrudescences so that accurate assessments of the failure rate can be made.

## CONCLUSIONS

Insufficient length of follow-up in antimalarial therapeutic assessments underestimates recrudescence rates and thus overestimates treatment efficacy. This can lead to continuation of a less efficacious drug or insufficient dosage, resulting in more treatment failures at the individual level and a higher risk of selecting resistance. This study suggests that the currently recommended length of follow-up of 28–42 days in therapeutic assessments may be insufficient for pregnant women, at least in low-transmission areas. Follow-up of 63 days should be considered for AL, and even longer might be needed for DP. The optimal duration of follow-up for pregnant women, particularly in high-transmission settings, requires further investigation.

## Supplementary Data

Supplementary materials are available at *Open Forum Infectious Diseases* online. Consisting of data provided by the authors to benefit the reader, the posted materials are not copyedited and are the sole responsibility of the authors, so questions or comments should be addressed to the corresponding author.

ofz264_suppl_supplementary_material
