# Supplementary material for: Optimal Duration of Follow-up for Assessing Antimalarial Efficacy in Pregnancy: A Retrospective Analysis of a Cohort Followed Up Until Delivery on the Thailand–Myanmar Border
Source: Open Forum Infect Dis. 2019 May 31;6(7):ofz264. doi: 10.1093/ofid/ofz264 (PMC6602886; doi:10.1093/ofid/ofz264)
Supplement: ofz264_suppl_supplementary_material [file ofz264_suppl_supplementary_material.pdf]

Supplement

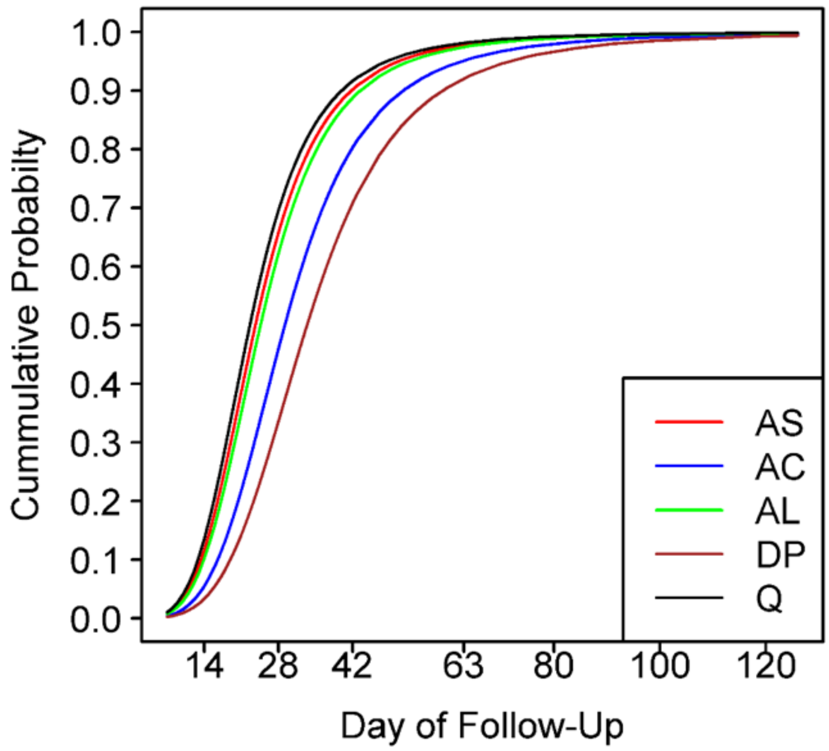

Supplementary Figure 1. Cumulative probability of recrudescence over time by treatment in pregnant women on the Thailand-Myanmar border, 1994–2010. AC, artesunate with clindamycin; AL, artemether-lumefantrine; AS, artesunate monotherapy; DP, dihydroartemisinin-piperaquine; Q, quinine monotherapy.

Supplementary analysis 1: a sensitivity analysis by the flexible parametric models

Using the variables in the final model, sensitivity analyses were conducted whether other parametric models gave a better fit to the data. Among the AFT models (without knots), log-logistic model was confirmed to have the smallest AIC (Supplementary Table 1). When Royston and Parmar's flexible parametric models (restricted cubic splines with knots) were applied, log-logistic model with 1 knot was with the smallest AIC. Because flexible parametric models lose the interpretability as AFT model, we preferred the original log-logistic model (without knots) as the final model. Instead, the prediction by log-logistic model with 1 knot was compared to our final model.

Graphically, the improvement of the fit to the data was mainly seen in the right-hand tail of the survival curve, particularly after 42 days (Supplementary Figure 2). The predicted cumulative probability of recrudescence (the proportion of recrudescence detected) at day 28, 42 and 63 were calculated based on this model (Supplementary Table 2). As expected from the graphical presentation, the gap between the final model and the sensitivity analysis model became wider when the follow-up days became longer. The predicted cumulative proportion of treatment failure detected by 42 or 63 were consistently lower by 1–7% than those by the final model.

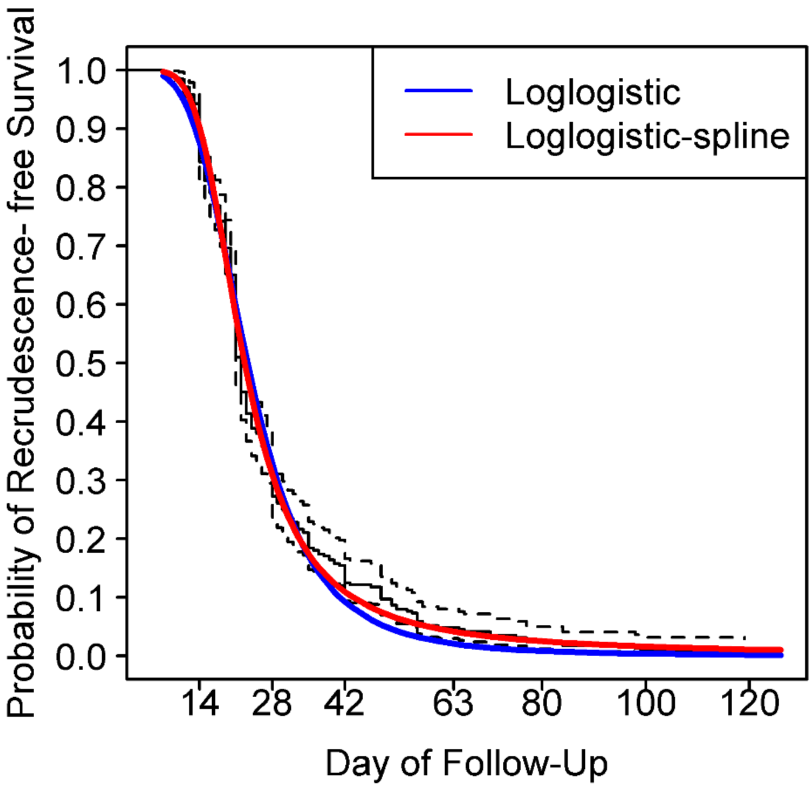

Supplementary Figure 2. Survival curves of multivariable model by log-logistic distribution model and splined log-logistic distribution model with one knot. Kaplan-Meier curve is shown with a black line with 95% confidence interval.

Supplementary Table 1. Akaike information criterion (AIC) comparing different accelerated failure time models and flexible parametric models with covariates.

Model	Number of knot	AIC
**Exponential**	0	857.3
**Weibull**	0	658.7
	1	504.7
	2	495.6
**Log normal**	0	525.5
	1	492.6
	2	493.4
**Log logistic**	0	513.8
	1	492.1
	2	493.5

AIC, Akaike information criterion.

Supplementary Table 2. Predicted cumulative proportions of treatment failure detected by days 28, 42 and 63 after antimalarial treatment by a model using log-logistic distribution with one knot.

Treatment	Predicted cumulative proportion of treatment failure detected by					
	Day 28		Day 42		Day 63	
	Proportion	95% CI	Proportion	95% CI	Proportion	95% CI
**Quinine**	0.71	0.67– 0.76	0.90	0.87–0.92	0.96	0.94–0.98
**AS**	0.67	0.55–0.78	0.88	0.81–0.93	0.95	0.92–0.98
**AL**	0.65	0.44–0.81	0.87	0.74–0.94	0.95	0.88–0.98
**AC**	0.49	0.29–0.69	0.78	0.60–0.89	0.91	0.80–0.96
**DP**	0.37	0.13–0.68	0.68	0.36–0.89	0.86	0.60–0.96

AC, artesunate with clindamycin; AL, artemether-lumefantrine; AS, artesunate monotherapy; CI, confidence interval; DP, dihydroartemisinin-piperaquine.

Supplementary analysis 2: a sensitivity analysis by a model without censoring by *P. vivax* infection

Using the final multivariable log-logistic model, another sensitivity analysis was conducted ignoring *P. vivax* intercalated infection. In this analysis, those who had *P. vivax* intercalated infection were not censored on the date of *P. vivax* intercalated infection, and the time to recrudescence was analysed for all episodes regardless of the *P. vivax* intercalated infection.

The predicted cumulative proportion of treatment failure detected by 28, 42, 63 days were consistently lower by 1–4% than those by the final model (Supplementary table 3).

Supplementary Table 3. Predicted cumulative proportions of treatment failure detected by days 28, 42 and 63 after antimalarial treatment by a model using log-logistic distribution without censoring *Plasmodium vivax* intercalated infection.

Treatment	Predicted cumulative proportion of treatment failure detected by					
	Day 28		Day 42		Day 63	
	Proportion	95% CI	Proportion	95% CI	Proportion	95% CI
**Quinine**	0.69	0.64–0.73	0.91	0.88–0.93	0.98	0.97–0.98
**AS**	0.62	0.50–0.74	0.88	0.81–0.93	0.97	0.95–0.98
**AL**	0.58	0.37–0.76	0.86	0.72–0.94	0.97	0.92–0.99
**AC**	0.42	0.24–0.64	0.77	0.58–0.89	0.94	0.86–0.97
**DP**	0.34	0.13–0.65	0.70	0.40–0.89	0.91	0.75–0.97

AC, artesunate with clindamycin; AL, artemether-lumefantrine; AS, artesunate monotherapy; CI, confidence interval; DP, dihydroartemisinin-piperaquine.

Supplementary analysis 3: a model including mefloquine monotherapy

A sensitivity analysis was conducted including mefloquine monotherapy (n=15), which is neither an artemisinin-based or quinine-based treatment. Mefloquine monotherapy data available for this additional analysis included episodes from 1994–1996 when high grade resistance to mefloquine was already prevalent in the area [1, 2].

In the univariable analysis, treatment was associated with time to recrudescence (not shown). With quinine as the reference, the time to recrudescence (95% confidence interval [CI]) was longer by 1·04-times (0·90 to 1·20) after AS, 1·08-times (0·86 to 1·35) after AL, 1.30-times (1·03 to 1·63) after AC, 1.46-times (1·02 to 2·08) after DP and 1·09-times (0·86 to 1·38) after mefloquine.

In the multivariable analysis, treatment was associated with time to recrudescence adjusted by baseline parasitemia. The adjusted interval to recrudescence was longer by 1·05-times (95% CI: 0·91 to 1·21) after AS, 1·09-times (0·87 to 1·36) after AL, 1·29-times (1·03 to 1·63) after AC, 1·48-times (1·04 to 2·12) after DP, and 1·08-times (0·86 to 1·37) after mefloquine than that after quinine.

The peak time of recrudescence was day 20 for quinine, day 21 for AS, AL and mefloquine, day 25 for AC and day 29 for DP.

The predicted cumulative proportions of recrudescence detected by commonly used follow-up days (day 28, 42 and 63) for the other drugs were almost unchanged by including mefloquine monotherapy, with differences of up to 1 % (0.01) (Supplementary Table 4).

Supplementary Table 4. Predicted cumulative proportions of treatment failure detected by days 28, 42 and 63 after antimalarial treatment including mefloquine monotherapy by a model using log-logistic distribution.

Treatment	Predicted cumulative proportion of treatment failure detected by					
	Day 28		Day 42		Day 63	
	Proportion	95% CI	Proportion	95% CI	Proportion	95% CI
**Quinine**	0.69	0.65–0.74	0.92	0.89–0.94	0.98	0.97–0.99
**AS**	0.66	0.53–0.76	0.90	0.84–0.94	0.98	0.96–0.99
**AL**	0.62	0.41–0.79	0.89	0.77–0.95	0.97	0.94–0.99
**AC**	0.46	0.26–0.67	0.80	0.63–0.91	0.95	0.89–0.98
**DP**	0.33	0.11–0.66	0.70	0.38–0.90	0.92	0.74–0.98
**Mefloquine**	0.63	0.41–0.80	0.89	0.77–0.95	0.97	0.94–0.99

AC, artesunate with clindamycin; AL, artemether-lumefantrine; AS, artesunate monotherapy; CI, confidence interval; DP, dihydroartemisinin-piperaquine.

References (Specific to Supplement)

1. McGready R, Cho T, Hkirijaroen L, et al. Quinine and mefloquine in the treatment of multidrug-resistant Plasmodium falciparum malaria in pregnancy. Ann Trop Med Parasitol **1998**; 92(6): 643-53.
2. Nosten F, Luxemburger C, ter Kuile FO, et al. Treatment of multidrug-resistant Plasmodium falciparum malaria with 3-day artesunate-mefloquine combination. J Infect Dis **1994**; 170(4): 971-7.
